# Concussion-Induced Oromandibular Dyskinesia

**DOI:** 10.7759/cureus.36075

**Published:** 2023-03-13

**Authors:** Brenda Zhang, Joseph P Settineri, Alan Chajet, Purushothaman Muthukanagaraj

**Affiliations:** 1 Medicine, Upstate Medical University at Norton College of Medicine, Syracuse, USA; 2 Psychiatry, SUNY (State University of New York) Upstate Medical University, Binghamton Clinical Campus, Binghamton, USA; 3 Psychiatry, United Health Services, Binghamton, USA

**Keywords:** orofacial movement, post-traumatic movement disorder, concussion, oromandibular, dyskinesia

## Abstract

We present a rare case of concussion-induced chronic oromandibular dyskinesia. The patient is a 51-year-old Caucasian male with complex medical history (including a history of multiple concussions) who presented to the emergency department for suicidal and paranoid ideation. At the time of the visit, the patient was noted to be exhibiting an oromandibular dyskinesia in the form of “teeth-chattering.” The first documented episode of his oromandibular dyskinesia dates back to a medical visit in December 2017. During this visit, the patient presented with teeth-chattering and tremors in his legs, hands, and head after a concussive event. Similar symptoms were noted by two different providers during two unrelated appointments one month later in January 2018. These symptoms were not mentioned in his records for four years and three months following the initial onset. They were noted again during an outpatient encounter for insomnia. During these four years, he was treated for a variety of conditions in both inpatient and outpatient settings, including gout, human immunodeficiency virus (HIV), stage four lymphoma, insomnia, and hepatitis C. Curiously, the dyskinesia symptoms reappeared several months after the completion of six cycles of etoposide, prednisone, vincristine, cyclophosphamide, and doxorubicin hydrochloride (R-EPOCH) chemotherapy in May 2021. Since reappearing, the symptoms have been worsening and significantly impacting the patient’s quality of life. Although concussion-induced dyskinesia has been previously described in the literature, this is to our knowledge the first described case of concussion-induced oromandibular dyskinesia.

## Introduction

Oromandibular dyskinesia has been widely understood to be involuntarily repetitive facial movements of the face or mouth such as lip smacking, chewing, or eye blinking [1]. Similar to the mechanism of tardive dyskinesia involving nigrostriatal inhibition of dopaminergic pathways, oromandibular dyskinesia is most commonly grouped into a list of extrapyramidal symptoms secondary to antipsychotic medication usage [2]. Specific oromandibular motor disorders incorporating muscles and cranial nerves involved in mastication have also been noted in motor disorders such as Parkinson’s disease, Bell’s palsy, or post-stroke paralysis [3]. Moreover, research has begun to shed light on post-traumatic brain injuries leading to movement disorders including but not limited to oromandibular dyskinesia [4,5].

There is little information in the literature detailing a correlation between multiple concussive injuries and the induction of oromandibular dyskinesia [6]. Furthermore, there is little to no information about chronically sustained oromandibular dyskinesia lasting years after the initial onset of movement was noted [7,8]. Therefore, we present a rare case of concussion-induced chronic oromandibular dyskinesia in a patient with no history of antipsychotic use. We specifically look through our patient’s history of concussions precipitated by contact sports and offer a potential mechanism of action explaining how aberrant neuronal regeneration might have contributed to a worsening of his continuous involuntary oromandibular dyskinesia long after the initial concussive insult.

## Case presentation

**Figure 1:**
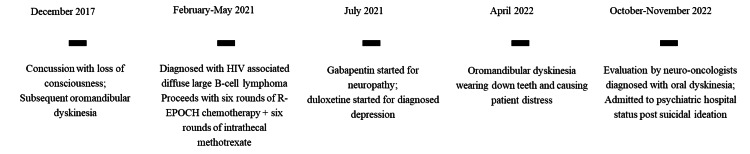
Patient timeline of oromandibular dyskinesia HIV: Human immunodeficiency virus; R-EPOCH chemotherapy: Regimen combination of rituximab, etoposide phosphate, prednisone, vincristine sulfate, cyclophosphamide, and doxorubicin hydrochloride.

A 51-year-old Caucasian male with a previous psychiatric history of depression voluntarily came to the emergency department for suicidal ideation and paranoia. He was also grappling with recurrent moderate major depressive disorder secondary to stage IV lymphoma. These factors in culmination led him to feelings of hopelessness and suicidal rumination, which prompted him to seek urgent medical help. The patient’s past medical history was significant for human immunodeficiency virus (HIV), stage four lymphoma, hepatitis C, shingles, cholecystitis, and multiple concussions from contact sports. It was noted that the patient first exhibited tremors and oromandibular dyskinesia described as “teeth-chattering” during a follow-up for a sports-related (pole vaulting) concussion in 2017 (Figure 1). The diagnosis of concussion was clinically made as the patient refused to undergo any type of head imaging due to his claustrophobia.

After being diagnosed with HIV-associated primary diffuse B-cell lymphoma in early 2021 and undergoing six total cycles of R-EPOCH chemotherapy along with six cycles of intrathecal methotrexate, his last chemotherapy cycle was completed in late May 2021. The patient was then prescribed gabapentin for the neuropathy and eventually began taking duloxetine for his depression in July 2021. The patient’s oral dyskinesia continued to worsen even after he completed his cycles of chemotherapy.

A year after all chemotherapeutic interventions were completed in May 2021, the patient continued to report dyskinesia that was detrimentally impacting his everyday life; his teeth had slowly begun to wear down, and his inability to have conversations without teeth-shaking interruptions between sentences became a point of frustrating annoyance. His primary care practitioner subsequently put him on a trial dose of clonazepam 0.5 mg in mid-October 2022 and referred him to neurology for evaluation a week later, where his clonazepam dose was increased from 0.5 to 1.5 mg daily. The patient's neuro-oncologist consulted with their movement disorder team for a second opinion, and they together came to the diagnosis of oromandibular dyskinesia. The patient was also asked to complete a computed tomography (CT) scan or magnetic resonance imaging (MRI) scan of the head many times by different providers over the years but refused. Subsequent follow-up was made with the patient to obtain permission to record his oromandibular dyskinesia; the following video was collected with the patient's permission to view and publicly distribute (Video 1). The Abnormal Involuntary Movement Scale (AIMS), typically used to assess the severity of dyskinesia, was calculated to be 23 for this patient, indicating severe dyskinesia.

**Video 1:** Oromandibular dyskinesia

Upon initial interview in the emergency department, the patient was alert and oriented albeit anxious. He spoke in a normal tone and rhythm with fast pressured speech. He exhibited psychomotor agitation with oral dyskinesia audible even while sitting across the room; this often frustrated him as it interrupted his ability to speak fluently. He stated that the “teeth-chattering” does not stop when he is awake and gets worse if he feels anxious or nervous. He reports that it began affecting his quality of life a few months after the initiation of his chemotherapy and continued throughout the day no matter what the weather, activity, or time of day was. His wife later noted that the teeth-chattering stops only when he sleeps.

Upon presentation, vitals showed a blood pressure of 135/91, pulse of 110, respiratory rate of 16, temperature of 36.3°C, and oxygen saturation of 99% on room air. Laboratory results showed a high glucose level at 125 mg/dL, slightly elevated albumin level at 5.1 g/dL, elevated total protein at 9.6 g/dL, and elevated uric acid level at 9.6 mg/dL. All other lab results were within normal limits.

During the next few days of inpatient hospitalization leading up to his discharge, the patient reported feeling less anxious after medication adjustments of discontinuing his previously prescribed clonazepam 1.5 mg and replacing it with trazodone 50 mg for sleep. At the time of discharge on day three of admission, the patient’s oromandibular dyskinesia, although still present and constant, was less intense and slower in rate.

## Discussion

Head injury and post-traumatic movement disorders are well-documented phenomena and have been reported in 13%-66% of patients. However, most of the documented cases of post-traumatic movement disorders have been recorded following severe injury resulting in a Glasgow Coma Scale of less than 8. Furthermore, the most common sequelae are in the form of tremors followed by dystonia and other movement disorders; oromandibular dyskinesia following concussion has not, to our knowledge, been documented in the literature [1].

Cases reporting oromandibular dystonia following a traumatic brain injury and dyskinesia following a concussion have some similar findings to this study [2,3]. However, both of these cases vary significantly from the current presentation. First, as described by Verma et al., we noticed a dyskinesia involving the teeth that resulted in a chattering noise rather than sustained dystonia [6]. Additionally, the onset of dyskinesia in our case was reported to be immediately after the concussive injury, whereas the oromandibular dystonia case by Verma et al. described the onset of symptoms three months after the injury. In the case of dyskinesia following concussion, Cottrill et al. described a young man who presented with startle myoclonus several months after a concussion [7]. Although consistent with the current study in the initial insult, the post-concussive sequelae differ drastically [2,3].

The current case is unique in the specific oromandibular dyskinesia that has been described in every chart review note as “teeth-chattering.” Notably, in addition to the unique mandibular dyskinesia, resting tremor as well as bilateral upper and lower extremity myoclonus were also present. It is important to note that while all of these symptoms were present immediately after the concussion, they worsened four years later to the extent where the patient had difficulty speaking full sentences and had noticeable dental wear.

The link between mild to severe head injury and movement disorders has not been extensively studied. Possible mechanisms include diffuse axonal injury with significant lesions to the cerebellar peduncle, the release of inflammatory cytokines creating reactive oxygen species causing subsequent damage, and possible genetic predisposition [1]. One of the reasons for the worsening of dyskinesia over the course of four years could be the regenerative nature of neurons, i.e., aberrant sprouting or increased sensitivity of neurons to neurotransmitters has been reported as a possible cause of a delayed presentation in post-traumatic movement disorders. In particular, Scott and Jankovic reported the mean presentation time for post-traumatic movement in adults to be 2.5 years [8].

Although we have to consider functional movement disorder as a differential diagnosis, it is less likely. While functional movement disorders are known for their variable presentations and time lengths, they fall under the category of conversion disorders [9]; this implies that there are specific triggers preceding the onset of the movement, which go away once the initial stressor is addressed [9]. This does not match the description of our patient, whose oromandibular dyskinesia is present regardless of the presence of stressors and has even worsened over time to the point of causing him distress.

Limitations of this case are the inability to objectively compare the severity of the dyskinesia from initial insult to present other than a subjective worsening described by the patient. Additionally, the lack of documentation from previous concussions prevented us from determining if this was the first presentation of post-concussive sequelae in this patient. This case report highlights the importance of handling concussion as a severe injury with significant sequelae. In this case, before the documented concussion with dyskinesia, tremor, and myoclonus, the patient reported 12 previous concussions. Documentation of previous concussions could have played a role in the presentation of the current case and should be noted when treating patients with a history of concussion. Further research utilizing neuroimaging studies, videos, and other diagnostic tools such as the AIMS to rule out differential diagnoses of functional movement disorders or tardive dyskinesia is warranted and would be useful in a case series study to supplement research in the future studies of oromandibular dyskinesia.

## Conclusions

Oromandibular dyskinesia is typically associated with long-term antipsychotic use or genetic neurodegenerative conditions, but it has also been noted as a consequence of other medications and diseases. Compared to the current literature available, we presented a rare case of chronic oromandibular dyskinesia status post concussion that further intensified years after the first onset of dyskinesia was noted. Further research into the possible mechanism of concussion-induced oromandibular dyskinesia would be crucial to select efficient treatment modalities to best alleviate lifestyle distress. Based on the case presented, oromandibular dyskinesia should thus be considered as a possible side effect in patients with a history of multiple contact sports-related head injuries.
